# Editorial: Engineering the Microbial Platform for the Production of Biologics and Small-Molecule Medicines

**DOI:** 10.3389/fmicb.2019.02307

**Published:** 2019-10-09

**Authors:** Dipesh Dhakal, Eung-Soo Kim, Mattheos Koffas

**Affiliations:** ^1^Department of Life Science and Biochemical Engineering, Sun Moon University, Asan, South Korea; ^2^Department of Biological Engineering, Inha University, Incheon, South Korea; ^3^Department of Chemical and Biological Engineering, Rensselaer Polytechnic Institute, Troy, NY, United States

**Keywords:** microbial cell factories, metabolic engineering, synthetic biology, heterologous production, biologics and small molecule medicines

Microorganisms are the prominent sources of valuable products ranging from large (e.g., proteins, carbohydrate polymers, nucleic acids, even cells) to small molecules (e.g., microbial metabolites, signaling molecules, growth factors, etc.). Most of these small molecules termed as “secondary metabolites (SM)s” are inessential to the producer for their growth and development. However, these SMs have significant applications in human and animal health (Demain, 2000; Bhan et al., 2013; Wang et al., 2016; Dhakal and Sohng, 2017). Besides, several biologics pharmaceutical ingredients extracted from animals, plants, and microorganisms such as antibodies, vaccines, receptor modulators or replacement/modulators of enzymes are applied for human welfare (Kinch, 2005; Lacana et al., 2007). The host microorganisms engineered for the production of such small molecular medicines or relatively complex biologics are termed as “microbial cell factories (MCF).” Recently, metabolic engineering approaches are developed for engineering of metabolism and biosynthetic pathways in these MCFs for better performance (Davy et al., 2017; Choi et al., 2018). The papers published in this Research Topic have attempted to explore the current state of the art of microbial engineering along with its diverse approaches.

Pham et al. have summarized the biological activities and applications of a variety of small molecular medicines and biologics. The manuscript has reviewed the diverse microbial systems used for the production of these biomolecules along with the versatile engineering strategies of such microbial platforms. Generally, each of the microbial strains can produce multiple compounds, but it can produce only subsets of these compounds under specific growth conditions. Therefore, variations in cultivation parameters can elicit the production and discovery of new SMs. For example, by changing cultivation parameters such as temperature, salinity, aeration, and even by altering the shape of the flasks, the production profile from a microbial platform can be altered (Bode et al., 2002). Pan et al. have provided comprehensive information regarding the exploration of structural diversity of microbe secondary metabolites using one strain many compounds (OSMAC) approach. They have presented the role of variation in medium, cultivation conditions, use of epigenetic modifiers, and co-cultivation in the discovery of novel secondary metabolites from diverse microbial sources utilizing OSMAC approach (Pan et al.).

*Escherichia coli* is reported as the most common cell factory for the production of both small molecules and biologics. The clear understanding of its physiological and genetic characteristics, fast-growth even in minimal salts medium, and availability of easy genetic manipulation techniques has established it as first-choice production host (Liu et al., 2015). Also, systems metabolic engineering approaches that combine knowledge of systems biology, synthetic biology, and evolutionary engineering into the traditional metabolic engineering, has facilitated the development of *E. coli* as a robust production host for heterologous expression of small molecules and complex biologics (Choi et al., 2019). So, different metabolic engineering approaches utilizing *E. coli* as microbial platform have been presented in this Research Topic. Wang et al. have reviewed different aspects of terpenoid production using *E. coli* including the metabolic engineering and genome engineering approaches. Li et al. utilized product resistance and targeted metabolic engineering for the production of equol in *E. coli*. Similarly, the combination of product tolerance, evolution engineering, and modular co-culture was utilized for the production of pinene (Niu et al.). The metabolic engineering approach utilizing gene over-expression cassette for enhanced production of nucleotide diphosphate (NDP)-sugars was utilized for generating the salicylate glucoside and other glycosylated variants (Qi et al.). Similarly, the gene-silencing approach was employed for enriching the titer of 3′-phosphoadenosine-5′-phosphosulfate (PAPS), which is donor substrate for sulfation of natural product (NP) precursors. Hence, by inhibiting the degradation of PAPS mediated by repression of PAPS reductase (*cysH*) and optimization of different sulfate donors significantly enhanced the production titer of naringenin-7-sulfate (Chu et al.). Ribosomally synthesized and post-translationally modified peptides (RiPPs) are special class of NPs with diverse structures and bioactivities, and thus possess a complex biosynthetic mechanism. Different aspects for heterologous expression of RiPPs in *E. coli* have been reviewed by Zhang et al.

Due to the presence of endotoxins in products obtained from Gram negative bacteria as *E. coli*, some of the non-lethal Gram-positive bacteria including the native producer strains such as actinobacteria or heterologous hosts [generally recognized as safe (GRAS)] such as *Bacillus* and *Corynebacterium* are used as excellent cell factories in industries. Actinobacteria are characterized as the most prominent producers of thousands of bioactive molecules, particularly small molecular medicines such as commercially available antibiotics and anticancer-drugs (Dhakal et al., 2017; Rangseekaew and Pathom-aree, 2019). In some cases, NPs from these actinomycetes are cryptic or not produced in a significant amount. Thus, precise metabolic engineering can be employed in a native host or genetically tractable alternative heterologous hosts for significant production. Li et al. performed whole genome sequencing of the producer strain, analyzed the genome data by computational tools and isolated nocardamine utilizing genome mining of *Streptomyces atratus* SCSIOZH16. Peng et al. used *S. lividans* as platform organism and optimized the host for higher heterologous expression of foreign biosynthetic gene cluster (BCG) by modulation by a number of global positive and negative regulatory genes, and genes encoding drug efflux pumps. Further the optimized strain was used for production of NPs of diverse nature such as actinorhodin, murayaquinone, hybrubins, piericidin A1, dehydrorabelomycin, and actinomycin D. Generally, the production of SMs in *Streptomyces* is controlled by a complex regulatory network that involves pathway-specific, pleiotropic, and global regulators, which tune the expression level of biosynthetic genes in response to a variation in diverse physiological and environmental conditions (van Wezel and McDowall, 2011). Hence, the engineering of such regulation cascades by activators and repressors have significant role in determining the productivity of target molecules. Yu et al. identified AdpAch, as a bidirectional pleiotropic regulator of natamycin biosynthesis in *S. chattanoogensis* L10. Subsequently, the production titer of natamycin was enhanced by mutating the AdpAch-binding sites, that had an inhibitory effect. Recently, the application of precise genetic engineering based on clustered regularly interspaced short palindromic repeats (CRISPER) and its associated protein (Cas9) has enabled the multiplexed genome engineering of actinomycetes including *Streptomyces*. Tao et al. have reviewed the application of CRISPR/Cas9 based genome editing in *Streptomyces* for discovery, characterization, and production of NPs. The recent advances in heterologous expression of RiPPs in *Streptomyces* have been presented by Zhang et al.

*Bacillus* species has an ability to adapt to varying environmental conditions and capacity for high production yield (Pham et al.), hence they are crucial industrial microorganisms. Further, the application of recent advances in metabolic engineering, enzyme/pathway engineering along with the synthetic biological tools have contributed to ameliorate the production titer from these microorganisms. Yang et al. utilized enzyme engineering of homogentisate dioxygenase for production of enhanced production of melanin. Similarly, a metabolic engineering approach was utilized for enhanced heterologous production of 2-deoxy-scyllo-inosose in *Bacillus subtilis*. Unlike *E.coli* and *Bacillus, Corynebacterium* has significant ability to utilize a variety of carbon sources (Heider and Wendisch, 2015). *C. glutanicum* is established as a major industrial producer of proteins, including biologics and enzymes as well as utilized in the production of diverse secondary metabolites as carotenoids, terpenes, and flavonoids. Lee and Kim have reviewed different crucial aspects of recombinant protein expression systems in *C. glutanicum* and its applications.

Fungi is the second largest kingdom of microorganism after bacteria. They are established as a promising source of bioactive natural products containing unique chemical compounds against various diseases (Singh et al., 2019). Ever since *Penicillium notatum* was identified as a source of penicillin, there has been immense interest in the exploration of the potential of fungal species for their capacity to produce versatile NPs with biotechnological and pharmaceutical applications. Guzmán-Chávez et al. have summarized on the engineering aspects of the *P. chrysogenum* for establishing it as a sustainable cell factory for NPs. They have provided the comprehensive summary about the basic biosynthetic logic of such NPs and various rational strategies for activation of biosynthetic gene clusters by optimizing culture parameters or targeted genetic engineering Guzmán-Chávez et al. In addition to the bacteria and fungi, the yeast strain such as *Saccharomyces cerevisiae* is successfully employed for the production of both bulk and fine chemicals (Kavšček et al., 2015). The different aspects of biosynthesis and prospects of metabolic engineering for the production of terpenoids in *S. cerevisiae* are summarized by Wang et al.

Taken together, all these papers illustrate the applicability of engineering of microbial platforms for the production of small molecular medicines to complex biologics. However, in case of all of these microbial cell factories (native, engineered, or heterologous) the industrial scale titer, yield, and productivity is generally difficult to achieve. The major constraint is unavailability of abundant information about their metabolic behavior, unavailability of appropriate genetic engineering tools, or complication in redesigning appropriate flux balance for diverting primary metabolites to target molecules (Bhan et al., 2013). Recently the application of large-scale genome sequencing, gene expression profiling, *in silico* metabolic modeling and simulation, and enzyme/pathway engineering has eased the rational approaches for metabolic engineering. Particularly, the traditional approach of single strain/pathway specific “try and test” approach is replaced by the application of systems metabolic engineering approach that utilizes integration of strain selection/development, pathway design/engineering, and enzyme selection/engineering for efficient production of target molecules. In addition, the application of tools for generating artificial genetic circuits/metabolic pathways incorporating efficient promoters, RBS, terminators, etc, or multiplexed genome engineering utilizing CRISPR/ Cas9 for gene knock-in/knock out, or activation/repression has advanced the engineering approaches of these MCFs to the next level. In future, it can be expected that it can be feasible to generate the super host with minimized genome and enriched metabolic pathway centered on particular class of molecules. Such super hosts can be engineered by introducing the synthetic genome to attain the designers' strain for specific target. The burgeoning development in both genetic studies as well as computational approaches such as artificial intelligence (AI) has great prospects for simulating the connection between the genomics and metabolomics to generate the intelligence in these super hosts, so that they can sense the environment condition, and respond rationally.

## Author Contributions

DD wrote the manuscript. E-SK and MK revised and corrected the manuscript. The final draft of the manuscript was finalized and approved for publication by all the authors.

### Conflict of Interest

The authors declare that the research was conducted in the absence of any commercial or financial relationships that could be construed as a potential conflict of interest.
